# DRD1 suppresses cell proliferation and reduces EGFR activation and *PD‐L1* expression in NSCLC

**DOI:** 10.1002/1878-0261.13608

**Published:** 2024-04-04

**Authors:** Christopher E. Grant, Amy L. Flis, Leila Toulabi, Adriana Zingone, Emily Rossi, Krist Aploks, Heather Sheppard, Bríd M. Ryan

**Affiliations:** ^1^ Laboratory of Human Carcinogenesis, Center for Cancer Research National Cancer Institute Bethesda MD USA; ^2^ Veterinary Pathology Core St. Jude Children's Research Hospital Memphis TN USA

**Keywords:** dopamine, DRD1, EGFR, non‐small cell lung cancer, PD‐L1

## Abstract

Dopamine (DA) acts in various key neurological and physiological processes as both a neurotransmitter and circulating hormone. Over the past several decades, the DA signaling network has been shown to regulate the progression of several types of solid tumors, and considerable evidence has shown it is a druggable pathway in the cancer cell context. However, the specific activity and effect of these pathway components appears to be tissue‐type and cell‐context‐dependent. In the present study, expression and methylation of dopamine receptor D1 (DRD1) were measured using RNA sequencing (RNAseq) and reverse transcription polymerase chain reaction (RT‐PCR) in non‐small cell lung cancer (NSCLC) samples, and validated using publicly available datasets, including The Cancer Genome Atlas (TCGA). *In vitro* and *in vivo* functional experiments were performed for cell proliferation and tumor growth, respectively. Mechanistic analyses of the transcriptome and kinome in DRD1‐modulated cells informed further experiments, which characterized the effects on the epidermal growth factor receptor (EGFR) pathway and programmed cell death 1 ligand 1 (PD‐L1) proteins. Through these experiments, we identified the *DRD1* gene as a negative regulator of disease progression in NSCLC. We show that DRD1, as well as other DA pathway components, are expressed in normal human lung tissue, and that loss of *DRD1* expression through promoter hypermethylation is a common feature in NSCLC patients and is associated with worse survival. At the cellular level, DRD1 affects proliferation by inhibiting the activation of EGFR and mitogen‐activated protein kinase 1/2 (ERK1/2). Interestingly, we also found that DRD1 regulates the expression of *PD‐L1* in lung cancer cells. Taken together, these results suggest that DRD1 methylation may constitute a biomarker of poor prognosis in NSCLC patients while other components of this pathway could be targeted to improve response to EGFR‐ and PD‐L1‐targeted therapies.

## Graphical abstract

DRD1 is expressed in normal lung respiratory epithelial cells, and its expression is reduced in NSCLC tumor tissues at least partially through promoter methylation. In lung cancer cells, DRD1 signaling reduces proliferation through reduced EGFR signaling, and it also reduces PD‐L1 expression. Impaired DRD1 signaling has the opposite effect.

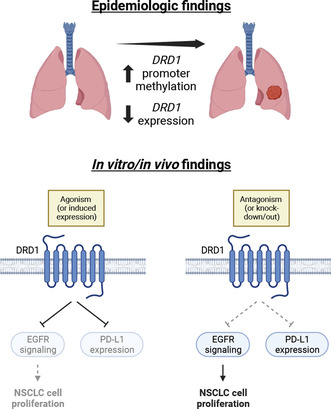

**Abbreviations**
CIS: lung carcinoma *in situ*
CNS: central nervous system
COMT: catechol‐*O*‐methyltransferase
CRISPR: clustered regularly interspaced short palindromic repeats
DA: dopamine
DAT1: dopamine transporter
DDC: dopa decarboxylase
ddPCR: droplet digital PCR
DR: dopamine receptor
DRD1: dopamine receptor D1
EGFR: epidermal growth factor receptor
ERK1/2: mitogen‐activated protein kinase 1/2
GPCR: G‐protein‐coupled receptor
IF: immunofluorescence
IHC: immunohistochemistry
LUAD: lung adenocarcinoma
LUSC: lung squamous cell carcinoma
MAO‐A and B: monoamine oxidase A and B
MAPK: mitogen‐activated protein kinases
NSCLC: non‐small cell lung cancer
PD‐L1: programmed death‐ligand 1
PI3K: phosphoinositide 3‐kinase
PLA: proximity ligation assay
RNAseq: RNA sequencing
RTKs: receptor tyrosine kinases
RT‐PCR: reverse transcription polymerase chain reaction
shRNA: short hairpin RNA
siRNA: small interfering RNA
TCGA: The Cancer Genome Atlas
TH: tyrosine hydroxylase

## 1 Introduction

Lung cancer is the leading cause of cancer‐related death both in the United States and worldwide [1]. In the last few decades, 5‐year relative survival rates improved slightly, from 14% to 19% [2]. One of the reasons for these dismal survival rates is that most patients present with advanced‐stage disease, a time at which systemic therapies are unlikely to be curative. Despite the introduction of second‐ and third‐generation cytotoxic drugs, as well as the use of immunotherapy and anti‐angiogenic therapies in combination with chemotherapy in the past decade, the median overall survival of advanced NSCLC has only marginally improved to 12–15 months [3]. Another key therapeutic advance in recent years has been the development of mutation‐targeting drugs against proteins such as EGFR and ALK [3]. However, resistance to these targeted therapies develops inevitably, and thus additional options are needed to continue treatment.

Dopamine (DA, 3‐hydroxytyramine) is a catecholamine neurotransmitter that has been extensively characterized in many vital central nervous system (CNS) processes, such as cognitive function, feeding, reward, attention, voluntary movement, sleep regulation, and sympathetic regulation [4]. Like other neurotransmitters, DA signals through a complex network of G‐protein‐coupled receptors (GPCRs), downstream effector molecules, metabolizing enzymes, and transporters that are present in both the CNS and the periphery.

DA receptors (DRs) are classified into two groups: D1‐like and D2‐like receptors [5]. D1‐like receptors (D1Rs) consist of DRD1 and DRD5, which generally associate with the Gα_s/olf_ subunit and activate adenylyl cyclase. Conversely, D2‐like receptors (D2Rs) consist of DRD2, DRD3, and DRD4, which typically partner with the Gα_i/o_ subunit and inhibit adenylyl cyclase activity [6, 7]. While best characterized in the CNS, DRs are also present in peripheral tissues including the heart and vasculature, eye, kidney, gastrointestinal tract, pancreas, and immune cells [8, 9, 10, 11].

Recent evidence also suggests peripheral DA signaling plays a role in cancer, affecting tumor growth and patient survival. Early evidence linking DRs and cancer came from target‐agnostic screens for drugs to repurpose as cancer therapies, and to date, at least nine unbiased screens have identified DR‐targeting compounds as potential antitumor therapies in multiple tumor types [12, 13, 14, 15, 16, 17, 18, 19, 20]. For example, one *in silico* screen using shRNA library‐identified gene signatures to find drugs that could synergize with EGFR inhibitor gefitinib in NSCLC identified thioridazine, an antipsychotic DRD2 antagonist, and showed that the two drugs synergistically induced cell death *in vitro* [15]. Stemming from these unbiased screens, as well as a growing body of literature suggesting D2Rs can function as tumor promoters, DRD2 antagonists have been investigated for efficacy in cancer treatment, and multiple D2R antagonists are currently in clinical trials [21].

In contrast, the roles of D1Rs in carcinogenesis are not as well described, but D1Rs have recently been identified as novel tumor suppressors in multiple cancers including breast cancer, glioblastoma, prolactinoma, endometrial cancer, and possibly gastrointestinal cancers, osteosarcoma, and liver cancers [22, 23, 24, 25, 26, 27, 28, 29, 30, 31, 32, 33, 34]. For example, in breast cancer, DRD1 agonists have been shown to inhibit breast cancer cell growth *in vitro* [23, 24], reduce cancer stemness and cell mobility *in vitro* [25], and reduce tumor growth [23] and lung metastasis [25] *in vivo*. In glioblastoma (GBM), DRD1 expression is associated with a more favorable prognosis [27], and one study found that DRD1 agonism inhibited cell growth through disrupting autophagic flux and showed that the DRD1 agonist SKF83959 and the chemotherapeutic temozolomide have a synergistic antitumor effect [27]. These studies point to a tumor suppressive role for D1Rs, but in other tumor types, the role of D1Rs is less clear as, for example, in both HCC and biliary tumors there are conflicting reports of DRD1's role [32, 33, 34, 35]. In summary, there is growing evidence for a tumor suppressive role for D1Rs in multiple tumor types, but more work is required to elucidate which tumor types and patient groups could benefit from therapeutic targeting of D1Rs. However, many DR‐targeting drugs are already FDA‐approved for treatment of neuropsychiatric disorders and hypertension, including D1R agonists, so the investigation of these medications for repurposing as cancer treatments represents a promising and potentially cost‐effective treatment strategy.

We recently demonstrated a link between a SNP in dopamine receptor D1 (*DRD1*) and risk of lung cancer [36]. Although we initially thought that the causal link between this SNP and lung cancer was nicotine exposure dependent via modulation of dopamine levels in the brain, we subsequently found that the relationship between this SNP and lung cancer persisted in never smokers, suggesting that a nicotine‐independent pathway could be involved. We therefore investigated the hypothesis that DRD1 is expressed in the lung and that it plays a role in lung carcinogenesis.

The present study demonstrates that DRD1 exerts a tumor suppressive‐like role in lung cancer cells via modulation of EGFR signaling and cell proliferation and that the dopamine network regulates the expression of the immune checkpoint molecule, programmed death‐ligand 1 (PD‐L1). These results raise the possibility that dopamine analogs could be repurposed to address critical unmet needs of lung cancer patients by augmenting efficacy and/or overcoming resistance to both anti‐EGFR and anti‐PD‐1 therapies.

## 2 Materials and methods

### 2.1 NCI‐MD case control study

Tissues for mRNA and IHC analysis were sourced from the NCI‐MD case control study. For the mRNA analysis, patients with histologically confirmed NSCLC and living in the Baltimore Metropolitan area were recruited to the ongoing NCI‐MD Case Control Study, as described previously [36], from seven Baltimore City hospitals: Baltimore Veterans Administration Medical Center, Bon Secours Hospital, MedStar Harbor Hospital, Sinai Hospital, Johns Hopkins Bayview Medical Center, The Johns Hopkins Hospital, and University of Maryland Medical Center. The collection of biospecimens and clinical and pathological information was approved by the UMD Institutional Review Board (IRB) for the participating institutions (UMD protocol #0298229). The research was also reviewed and approved by the NCI IRB (OH98‐C‐N027). This study was conducted in accordance with the Declaration of Helsinki, and written informed consent was obtained from all patients. Summary characteristics of cases and controls are shown in Table S1. Fresh sections of human lung tumor tissues and adjacent normal lung tissues were obtained from patients directly after surgery. After surgical removal, each tumor was macroscopically dissected by a pathologist to remove normal tissue. FFPE H&E sections were used to confirm the tumors contained mostly tumor cells and to inspect nonadjacent normal tissues. Each tissue was transferred to a sample collection tube, flash frozen, and stored at −80 °C. Specimens were transported to the NCI on dry ice within 24 h and stored at −80 °C until molecular analyses were performed.

Immunohistochemistry was performed on tissues collected from immediate autopsy. Immediate autopsy samples for analysis of the dopamine network in normal samples were obtained from the University of Maryland (UMD) hospital, which is part of the NCI‐MD study population. These tissues were received frozen from the UMD biorepository, cut, and paraffin‐embedded for analysis.

### 2.2 DRD1 methylation assessment in preinvasive lesions

Analysis of *DRD1* gene methylation in preinvasive lung lesions was performed using data obtained and described in a recent study [37]. Briefly, patients with lung carcinoma *in situ* (CIS), the preinvasive form of squamous cell carcinoma (LUSC), completed sequential biopsies, and were followed longitudinally. Researchers utilized laser‐capture microdissection to obtain the CIS biopsy lesion. DNA was extracted and used for genome‐wide methylation sequencing to assess early markers of disease progression in lesions of LUSC patients that progressed to invasive tumors (*n* = 22), lesions that regressed to normal (*n* = 13), and histologically verified control tissue (*n* = 20).

Raw idat files were downloaded from GSE108124 and imported into the r statistical programming environment (version 1.2.1335) using the bioconductor package minfi (version 1.30.0). The data were normalized using the functional normalization method [38]. We identified 12 CpGs with *DRD1* as the closest gene based on the *IlluminaHumanMethylation450kanno.ilmn12.hg19* annotation package (Table S2).

### 2.3 Immunohistochemistry

Immunohistochemical (IHC) analysis was completed using the Leica bond system (Agilent, Santa Clara, CA, USA; cat. #22.2201). In brief, we stained formalin‐fixed paraffin‐embedded tissue specimens harvested from the lungs of autopsy patients that died of causes other than lung cancer as part of the NCI‐MD Case Control Study. Human adrenal (cat. #HP‐501) and kidney (cat. #HP‐901) paraffin sections were acquired from Zyagen (San Diego, CA, USA) and used as positive control tissues based on recommendation from primary antibody datasheet provided by manufacturers. Human brain basal ganglia paraffin sections were acquired from Novus Biologicals Inc (cat. #NBP2‐77751; Centennial, CO, USA) as an additional positive control tissue. The tissue was mounted on glass slides and deparaffinized in xylene. The tissues were then gradually hydrated in a series of increasingly diluted ethanol baths concluding with pure DI water. Tissue was then neutralized using a 20‐min incubation in a 3% peroxide bath. Next, antigen retrieval was performed in a steam bath while slides incubated in pH 6 citrate antigen retrieval buffer for 15 min (Agilent Dako, Santa Clara, CA, USA, cat. #S203130‐2).

Following antigen retrieval, slides were incubated overnight at 4 °C in primary antibody or species‐matched IgG control antibody diluted with Antibody Diluent (Agilent Dako, cat. #S3022). See Table 1 for a complete list of primary antibodies and preparations used for IHC in this study. After washing slides in PBS‐T (PBS with 0.02% Tween‐20) with gentle shaking three times for 5 min, the slides were then incubated with the appropriate biotinylated secondary antibody (Agilent Dako, cat. #E0354 for rabbit IgG and cat. #E0432 for mouse IgG; Vector PK‐6104 for rat IgG). The stained tissue was then further conjugated with the VectaStain ABC Peroxidase kit to enhance signal sensitivity (Vector Laboratories, Newark, CA, USA, cat. #PK‐4000). The fully conjugated slides were then developed by incubation for an appropriate time in 3,3′‐Diaminobenzidine (DAB) (Agilent Dako, cat. #K3468). Lastly, the developed slides were counterstained with Hematoxylin (Agilent Dako, cat. #CS700) for 2 min.

**Table 1:** Primary antibodies used for IHC analysis of DA pathway in lung tissue.

Antigen	Manufacturer	Cat #	Dilution
DRD1 antibody #1	Calbiochem	Ab9141	1 : 200
DRD1 antibody #2	Sigma	D2944	1 : 500
DRD2	Millipore	AB5084P	1 : 100
DRD3	Abcam	ab42114	1 : 500
DRD4	Abcam	ab135978	1 : 100
DRD5	Santa Cruz	sc‐376088	1 : 100
MAO‐A	Abcam	ab126751	1 : 100
MAO‐B	Abcam	ab125010	1 : 25
COMT	Abcam	ab113521	1 : 300
DAT1	Novus	mAb16	1 : 100
TH	Abcam	ab112	1 : 300
DDC	Abcam	ab211535	1 : 500
Rabbit IgG Control	Invitrogen	31235	Varies^a^
Mouse IgG Control	Invitrogen	31903	Varies^a^
Rat IgG Control	Invitrogen	14–4321‐82	Varies^a^

^a^ Calculated to match primary antibody concentration.

The fully stained slides were then dehydrated in a series of increasingly potent ethanol baths concluding with xylenes. The dehydrated slides were then covered with Toluene mounting medium and immediately cover‐slipped. Mounted slides were imaged on an Olympus BX40 microscope (Olympus, Tokyo, Japan) using cellsens software or using aperio imagescope software.

### 2.4 Immunofluorescence

Immunofluorescent (IF) analysis was performed on fixed cells, grown on uncoated Lab Tek II chambered slides (ThermoFisher, Waltham, MA, USA, cat. #154334). Cells (1 × 10^4^ to 5 × 10^4^/well) were seeded in each chamber and cultured under appropriate experimental conditions for 24–72 h. Then, cells were washed with PBS and fixed in 4% paraformaldehyde for 15 min. Cells were then thoroughly washed in excess PBS, at least four times, to prevent over‐fixation. In many cases, cells were then permeabilized in a blocking buffer consisting of 0.3% Trition‐100, 2% bovine serum albumin (BSA) (Sigma, St. Louis, MO, USA, cat. #A9418), and 10% normal donkey serum (Sigma, cat. #D9663). Then, cells were stained in a buffer composed of blocking buffer without any Triton‐100. Cells were then incubated overnight at 4 °C in the following primary antibody diluted in staining buffer: PDL1 (E1L3N) (Cell Signaling, Danvers, MA, USA, #13684, 1 : 400) or for Fig. S5C only Abcam (Cambridge, MA, USA, ab205921, clone 28‐8, 1 : 400), followed by washing and 1 h incubation with Alexa Fluor 488 secondary antibody (Invitrogen, Waltham, MA, USA; #A‐21206, 1 : 400). All washes in between staining steps were done for 10 min using staining buffer.

After all staining and subsequent wash steps, polystyrene chamber walls were removed and cells were covered with roughly 200 μL of Vectashield mounting medium containing DAPI (Vector, Newark, CA, USA). Slides could then be imaged on a confocal microscope as quickly as 5 min after mounting, and up to 30 days later if stored at −20 °C. Images were collected using the Zeiss Airyscan LSM800 confocal microscope (Carl Zeiss AG, Oberkochen, Germany).

### 2.5 Cell lines

H727 (RRID:CVCL_1584), H1299 (RRID:CVCL_0060), A549 (RRID:CVCL_0023), and H292 (RRID:CVCL_0455) cells were purchased from American Tissue Culture Collection (Manassas, VA, USA) and periodically underwent short tandem repeat profiling (Frederick National Laboratory for Cancer Research), and verified in STR database, to authenticate their identity every 1–2 years. The cell lines have the following characteristics: H727, bronchial carcinoid tumor, KRAS (G12V) and p53 (Q165_S166insYKQ) mutations; A549, lung adenocarcinoma, KRAS (G12S) mutation; H1299, lung large cell carcinoma, p53 null and NRAS (Q61L) mutations; and H292, lung mucoepidermoid carcinoma [39, 40, 41]. Cells also underwent periodic mycoplasma testing to ensure that all experiments were performed with mycoplasma‐free cells. All cell lines were cultured in RPMI 1640 Medium (Gibco, cat. #11875093; Thermo Fisher Scientific, Waltham, MA, USA) supplemented with 10% charcoal‐stripped fetal bovine serum (FBS) (Gibco, cat. #12676029; Thermo Fisher Scientific), 1% penicillin/streptomycin and 2 mm l‐glutamine. Cells were grown at 37 °C under 5% CO_2_.

### 2.6 Plasmids details

pLX304‐V5‐Blast‐Empty vector (Lentiviral Gateway(R) destination vector with a blasticidin resistance marker and a V5 epitope tag) and pLX304‐V5‐Blast‐DRD1 vector were purchased from TransOMIC technologies. H1299 and H292 cells were transfected with pLX304‐V5‐Blast‐DRD1 or vector followed by selection with blasticidin to generate stable cell lines. Several single colonies were picked and expanded. DRD1 overexpression was confirmed by western blotting and immunofluorescence.

Sherwood UltramiR shRNA_control and sherwood UltramiR shRNA_DRD1 constructs were purchased from TransOMIC technologies. To generate stable DRD1 knock down cell lines, H727 and A549 cells were transfected with DRD1‐targeted shRNA and control shRNA followed by selection with puromycin. DRD1 knock down was confirmed by quantitative RT‐PCR.

pX458_Cas9‐2A‐eGFP_DRD1‐IVT‐773 and pX458_Cas9‐2A‐eGFP_DRD1‐IVT‐774 are bacterial plasmids each encoding Cas9 and a gRNA targeting exon 1 of the *DRD1* gene. These vectors were co‐transfected into H727 and A549 cells to eliminate expression of DRD1 using CRISPR. Following transfection, cells were selected for GFP expression using flow cytometry.

### 2.7 siRNA knockdown of DRD1

siRNA targeting DRD1 (cat. #4392421, siRNA ID s4281, s4282, and s4283) and nontargeted control siRNA (cat. #4390844) were purchased from Invitrogen. To transiently knock down DRD1, H727 cells were transfected with DRD1‐targeted siRNA or negative control siRNA using Lipofectamine 3000 (Thermo Fisher Scientific) according to manufacturer's protocol. Briefly, cells were plated at 1.2 × 10^6^ per 100 mm dish or 2 × 10^5^ per well of a 6‐well plate. The following day, siRNA oligomers (10 μm) were diluted in Opti‐MEM reduced serum medium and mixed with an equal volume of Lipofectamine 3000 pre‐diluted in Opti‐MEM. After 15 min incubation at room temperature, the complexes in Opti‐MEM were added to the cells for a final siRNA concentration of 15 nm. DRD1 knock down was confirmed by ddPCR following RNA extraction with RNeasy Plus Mini Kit (Qiagen, Venlo, The Netherlands, cat. #74134) and cDNA synthesis with iScript Select cDNA Synthesis Kit (Bio‐Rad, Hercules, CA, USA, cat. #1708897).

### 2.8 Cell proliferation assays

To quantitatively measure cell proliferation, we used the xCELLigence RTCA DP instrument (Roche Diagnostics, Mannheim, Germany), as per the manufacturer's instructions. Cells (1 × 10^3^ to 5 × 10^3^/well) were seeded in an E‐plate in appropriate medium (Roche Diagnostics). Cell indices were measured every 15 min for up to 120 h. Each treatment condition was measured in four replicates.

### 2.9 Quantitative RT‐PCR

RNA was isolated using Trizol Reagent (Medical Research Center Inc., Cincinnati, OH, USA, cat. #TR 118). cDNA was prepared using the ABI High Capacity kit (Thermo Fisher Scientific) with 500–1000 ng of genomic DNA‐free total RNA. PCR reactions were carried out with TaqMan Gene Expression assay probes (Thermo Fisher Scientific) for DRD1 (Hs00265425) and PD‐L1 (Hs00204257) mRNA. The data were acquired using the Applied Biosystems abi sds 2.4 Software Package (Thermo Fisher Scientific) and analyzed using the $2^{-\Delta \Delta C_{t}}$ method. The *C*_t_ value of these genes was normalized by subtracting the *C*_t_ of the endogenous control 18S or beta‐2‐microglobulin (B2M) mRNA.

### 2.10 Droplet digital PCR

RNA was isolated using RNeasy Plus Mini kit (Qiagen, cat. #74134). RNA purity and concentration was evaluated with spectrophotometer (Denovix DS‐11), and RNA concentration and RINe values were determined using Agilent TapeStation RNA ScreenTape. cDNA was prepared using the iScript Select cDNA Synthesis kit (Bio‐Rad, cat. #1708896) using oligo‐(dT) primers with 500–1000 ng of genomic DNA‐free total RNA. Digital droplet PCR (ddPCR) gene expression assay probes for DRD1 (dHsaCPE5042312) and ESD (dHsaCPE5037365) were obtained from Bio‐Rad. Approximately 11 μL containing 10–50 ng of cDNA and 250 nmol·L^−1^ probes per sample were dispensed in a 96‐well PCR plate. Another 11 μL containing Droplet PCR Supermix (Bio‐Rad) was added for a final volume of 22 μL. Droplet generation was performed using the QX200 Automated Droplet Generator (Bio‐Rad). After droplet generation, the plate was sealed with a pierceable foil seal at 180 °C for 5 s using the Bio‐Rad PX1 Plate Sealer (Bio‐Rad) and placed into a C1000 Touch Thermal Cycler with 96‐Deep Well Reaction Module (Bio‐Rad) using the following PCR conditions: 95 °C for 10 min followed by 40 cycles of denaturation at 94 °C for 30 s, annealing and extension at 55 °C for 1 min, enzyme deactivation at 98 °C for 10 min and a final ramp down and hold at 4 °C. Plates were then loaded and droplets counted using the Bio‐Rad QX200 Droplet Reader (Bio‐Rad). Data were analyzed using qx manager (Bio‐Rad).

### 2.11 Phospho‐kinase array analysis

To screen which receptor tyrosine kinases (RTKs) are targeted by DRD1, the Human RTK Phosphorylation Antibody Array Kit (R&D human phosphor‐kinase array, Cat No.: ARY003B) was used as a quick, sensitive, and inexpensive screening tool to simultaneously detect the relative site‐specific phosphorylation of 43 kinases and 2 related total proteins. DRD1‐manipulated cell lines (OE and shRNA KD) and the respective control cells were grown in culture until reaching 70–80% confluence. Then cell lysates were harvested using lysis buffer containing protease and phosphatase inhibitors, and the supernatants were used as whole‐tissue lysates and processed according to the manufacturer's protocol. The membrane blots were developed, and images were acquired. The levels of phosphorylation of the duplicated spots were quantified using imagej (Bethesda, MD, USA) to compare phosphorylation of the manipulated cells compared to respective controls. Values represent the mean of duplicate spots for each protein.

### 2.12 Western blotting

Western blotting analysis was completed using the Bio‐Rad Turbo Blot system (cat. #1704150). Briefly, cells were washed with PBS and then lysed with an appropriate volume of RIPA cell lysis buffer supplemented with Halt™ Protease and Phosphatase Inhibitor Cocktail (ThermoFisher, cat. #78441). After an incubation of 5 min on ice, cells were scraped and pipetted into 1.5 mL Eppendorf tubes and then centrifuged for 10 min at 14 000 ***g*** at 4 °C. The supernatant was collected and either frozen at −80 °C or processed further immediately. Protein concentration was measured using Pierce™ BCA Protein Assay Kit (ThermoFisher, cat. #23227).

Samples were boiled for 10 min at 99 °C, after adding 1/3 volume of denaturation buffer consisting of Laemmli loading buffer (Bio‐Rad, cat. #161‐0747) mixed with 10% β‐mercaptoethanol. The denatured samples were then loaded and electrophoresed through a 4–20% polyacrylamide gel in SDS Tris‐glycine running buffer. Then, proteins were transferred to a polyvinylidene difluoride membrane (PVDF) membrane using the BioRad Turbo Blot transfer machine (Bio‐Rad, cat. #1704150). Membranes were incubated overnight at 4 °C with the following primary antibodies, all diluted in Superblock (Thermo Scientific, #37515): EGFR (Cell Signaling, #4267S, 1 : 1000), pEGFR cocktail (Cell Signaling, #3777S and #2220S, both 1 : 1000), ERK1/2 (Cell Signaling, #4695T, 1 : 1000), pERK1/2 (Cell Signaling, #4370T, 1 : 1000), PDL1 (E1J2J(TM)) (Cell Signaling, #13684A, 1 : 1000), PDL1 (E1L3N) (Cell Signaling, #13684, 1 : 1000), V5 (Invitrogen, #R960‐25, 1 : 1000), DRD1 (Sigma, #D2944, 1 : 1000), GAPDH (Sigma‐Aldrich, #MAB374, 1 : 10 000), and actin (Abcam, #ab328, 1 : 5000); followed by washing with TBST and incubating for 1–2 h at room temperature with one of the following secondary antibodies: anti‐mouse HRP‐linked IgG (Cell Signaling, #7076S), anti‐rabbit HRP‐linked IgG (Cell Signaling, #7074S), or anti‐rat peroxidase AffiniPure donkey IgG (Jackson ImmunoResearch Laboratories, Inc., West Grove, PA, USA, #712‐035‐150). All Western blots were imaged using the BioRad ChemiDoc Touch Imaging System, and bands were quantified using imagelab software (Bio‐Rad).

### 2.13 Proximity ligation assay

Proximity ligation assay (PLA) was performed on fixed cells grown in uncoated ibidi μ‐Slide 8 Well Glass Bottom chambered coverslips (cat. #80827). Cells (1.5 × 10^4^/well) were seeded in each chamber to achieve 50–70% confluency and the following day were treated with serum‐free media for 4 h with or without EGF stimulation (25 ng·mL^−1^) for 10 min. Cells were washed with PBS and fixed with 4% paraformaldehyde for 10 min, followed by three additional PBS washes. PLA was performed according to kit protocol using the Sigma‐Aldrich Duolink *In Situ* PLA Probe Anti‐Rabbit PLUS (cat. #DUO92002), Duolink *In Situ* PLA Probe Anti‐Mouse MINUS (cat. #DUO92004), and Duolink *In Situ* Detection Reagents Red (cat. #DUO92008) kits. Briefly, each well was blocked with 2–3 drops of Duolink Blocking Solution for 1 h in a humidity chamber at 37 °C, then incubated overnight in a humidity chamber at 4 °C with 150 μL per well of primary antibody diluted in Duolink Antibody Diluent. Primary antibodies were rabbit EGFR (1 : 100, Cell Signaling, #4267) plus mouse EGFR (1 : 15, Santa Cruz, Dallas, TX, USA, sc‐377229) or rabbit EGFR plus mouse V5 tag (1 : 250, Thermofisher P/N46‐0705). Following overnight antibody incubation, antibody solution was tapped off, and wells were washed with Wash Buffer A twice for 5 min. PLA probe solution composed of PLUS and MINUS probes each at 1 : 5 in the Antibody Diluent was added for a 1‐h incubation in a humidity chamber at 37 °C. Wells were washed twice for 5 min with Wash Buffer A. Then a ligation solution, composed of 5× Duolink Ligation buffer diluted 1 : 5 in high purity water with Ligase added at 1 : 40, was added for a 30‐min incubation in a humidity chamber at 37 °C. Wells were again washed twice for 5 min with Wash Buffer A. Amplification solution, composed of 5× Amplification buffer diluted 1 : 5 in high purity water plus Polymerase added at a 1 : 80 dilution, was added for a 100‐min incubation at 37 °C in a humidity chamber wrapped in foil. Wells were then washed twice for 10 min with 1× Wash Buffer B and once for at least 1 min with 0.01× Wash Buffer B. DAPI staining was performed using a 5 min incubation with Spectral DAPI (FP1490) at 1 drop per 500 μL, followed by three 5‐min washes with 0.01× Wash Buffer B. Buffer was tapped off, and cells were mounted with ibidi mounting medium without DAPI (cat. #50001). Wells were imaged as z‐stacks along an automated meander pattern using a Nikon SoRa Spinning Disk microscope, and PLA signals were quantified by manually selecting the first 20 complete cells (at least 20 cells from at least 5 z‐stacks) per well from the captured z‐stacks and quantifying the number of PLA signals per cell with imaris software (Oxford Instruments, Zurich, Switzerland).

### 2.14 Animal studies

NU/NU nude mice were obtained from Charles River (Crl:NU‐Foxn1^nu^; strain code 088) and housed in the NCI animal facility. Only female mice (approximately 4–5 weeks of age) were used for all experiments. Mice were kept under the pre‐approved guidelines within NCI. Mice were housed in pathogen‐free conditions. Additional information (i.e. sample size, replicates) is described in figure legends.

Mice were subcutaneously injected with 100 000 H727 Pooled Ctrl or H727 DRD1 KO cells suspended in a solution of 50% Matrigel and 50% RPMI 1640 cell culture media. Each mouse was bilaterally injected with the two cell lines on separate flanks. Mice were considered “end stage” when the tumor reached 18 mm in at least one dimension. Tumor growth was monitored three times per week by measuring length and width.

All protocols used for animal experiments in this study were approved by the NCI‐Bethesda ACUC Guidelines/Policies (ACUC No. LHC‐009‐2).

## 3 Results

### 3.1 The dopamine network is present in normal human lung at the protein level

**Fig. 1:**
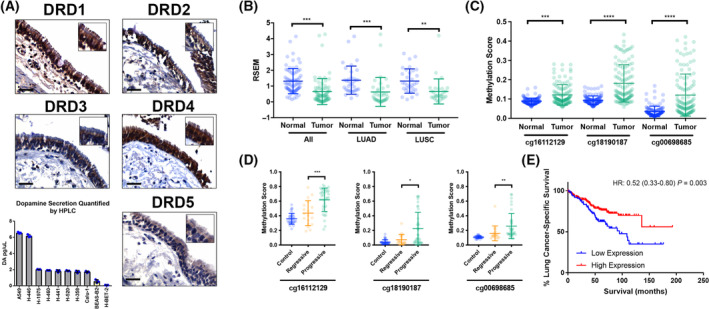
DRD1 expression in normal and malignant lung tissue. (A) Immunohistochemistry‐based expression of dopamine receptors 1–5 in normal human bronchial tissues and levels of dopamine produced by lung cell lines. Scale bars = 50 μm for images captured at 200× magnification. Enlarged area captured at 400× magnification. Yellow boxes denote non‐transformed cells, gray boxes denote cancer cell lines. (B) Expression of DRD1 mRNA by RNAseq data in the NCI‐MD study in normal (non‐involved adjacent) and tumor tissues. (C) Methylation levels of probes in the *DRD1* promoter in the NCI‐MD study in normal (non‐involved adjacent) and NSCLC tumor tissues. (D) Methylation levels of probes in the *DRD1* promoter in preinvasive lesions that progress to LUSC or regress. (E) Relationship between DRD1 mRNA expression and lung cancer‐specific survival in stage I LUAD patients in the NCI‐MD study. Statistical significance in panels B, C, and D determined using two‐tailed *t*‐tests; significance in E determined using Cox regression. LUAD denotes lung adenocarcinoma, LUSC denotes lung squamous cell carcinoma, and RSEM denotes RNASeq by Expectation–Maximization. Graphs in panels A, B, C, and D show mean ± SD. *P < 0.05, **P < 0.01, ***P < 0.001, ****P < 0.0001.

To understand the relevance of the dopamine network in the lung, we first characterized proteins related to dopamine function in normal human bronchial tissue using lung tissues from donors that were collected immediately after autopsy via the NCI‐MD case control study. As shown in Fig. 1, both class 1 (DRD1/DRD5) and class 2 (DRD2, DRD3, DRD4) dopamine receptors are expressed in normal human lung tissue (Fig. 1A, Fig. S1A). Expression of all five DRs was observed in the membrane and cytoplasm of respiratory epithelial cells, glandular epithelial cells, and to a lesser degree interstitial/stromal cells. An additional antibody for DRD1 was used to validate the DRD1 immunostaining and similarly showed expression of DRD1 in respiratory epithelium, in serous glandular epithelium, and sparsely in stromal cells (Fig. S1B). Interestingly, we observed that both DRD1 and DRD4 expression was enriched on bronchial cilia (Fig. 1A). IHC data also indicate that the DA metabolizing enzymes monoamine oxidase A and B (MAO‐A and B) and catechol‐O‐methyltransferase (COMT) are present in respiratory epithelial cells (Fig. S1A), as are enzymes involved in dopamine synthesis, including dopa decarboxylase (DDC) and tyrosine hydroxylase (TH) (Fig. S1A). Further, we detected expression of DAT1, the dopamine transporter responsible for shuttling dopamine into the cell (Fig. S1A). Finally, high‐performance liquid chromatography (HPLC) analysis detected dopamine secretion from various lung cancer cell lines grown *in vitro* (Fig. 1A) while RNAseq analysis of patients in the NCI‐MD study showed that transcript levels of both TH and DDC, key enzymes involved in the conversion of tyrosine to dopamine, are increased in lung cancer, suggesting a mechanism by which dopamine production is increased in lung cancer cells (Fig. S2J).

### 3.2 DRD1 is hypermethylated and downregulated in lung cancer

Our previous work showed that *DRD1* is a lung cancer susceptibility gene [36]. Therefore, to investigate the hypothesis that DRD1 is directly involved in lung cancer, we measured DRD1 mRNA expression in both human lung cancer tissues and paired non‐involved tissues in samples from the NCI‐MD case control study using RT‐PCR. We found DRD1 mRNA expression significantly downregulated by ~80% in human lung cancer tissues as compared with non‐involved tissues (Fig. S2A). This observation was validated in an independent set of samples using RNAseq from NCI‐MD (Fig. 1B) and from TCGA data for lung adenocarcinoma (LUAD) and squamous cell carcinoma (LUSC) (Fig. S2B,C). As the promoter of *DRD1* lies in a CpG island (Fig. S2H), we assessed whether the downregulation of *DRD1* corresponds with increased methylation of the *DRD1* promoter in tumor versus normal tissue. Indeed, we found that the *DRD1* promoter is significantly hypermethylated in lung cancer using samples from the NCI‐MD cohort (Fig. 1C) and then validated this observation using squamous cell carcinoma and adenocarcinoma data from TCGA (Fig. S2D,E). Furthermore, we found a negative correlation between DRD1 mRNA expression and methylation (Fig. S2F,G). Treatment of lung cancer cells with the demethylation agent 5′AZA increased mRNA expression of DRD1, further supporting the epigenetic regulation of *DRD1* expression in lung cancer (data not shown). Additionally, in a cohort of patients with preinvasive carcinoma *in situ* (CIS), we observed increased methylation of the *DRD1* promoter in preinvasive lesions that progress to LUSC compared to lesions that regress (Fig. 1D, Fig. S2I). Combined, these data suggest that *DRD1* gene methylation controls DRD1 expression in lung cancer and that aberrant *DRD1* methylation is an early event in lung tumorigenesis. Interestingly, we also found that decreased DRD1 expression coupled with increased methylation of its promoter is observed across several tumor types (Fig. S2K,L) and that high DRD1 expression is associated with better prognosis in stage I lung adenocarcinoma (Fig. 1E). Collectively, our data point toward a poor prognostic role for loss of DRD1 expression and increased *DRD1* methylation in cancer.

### 3.3 DRD1 modulates cell proliferation

**Fig. 2:**
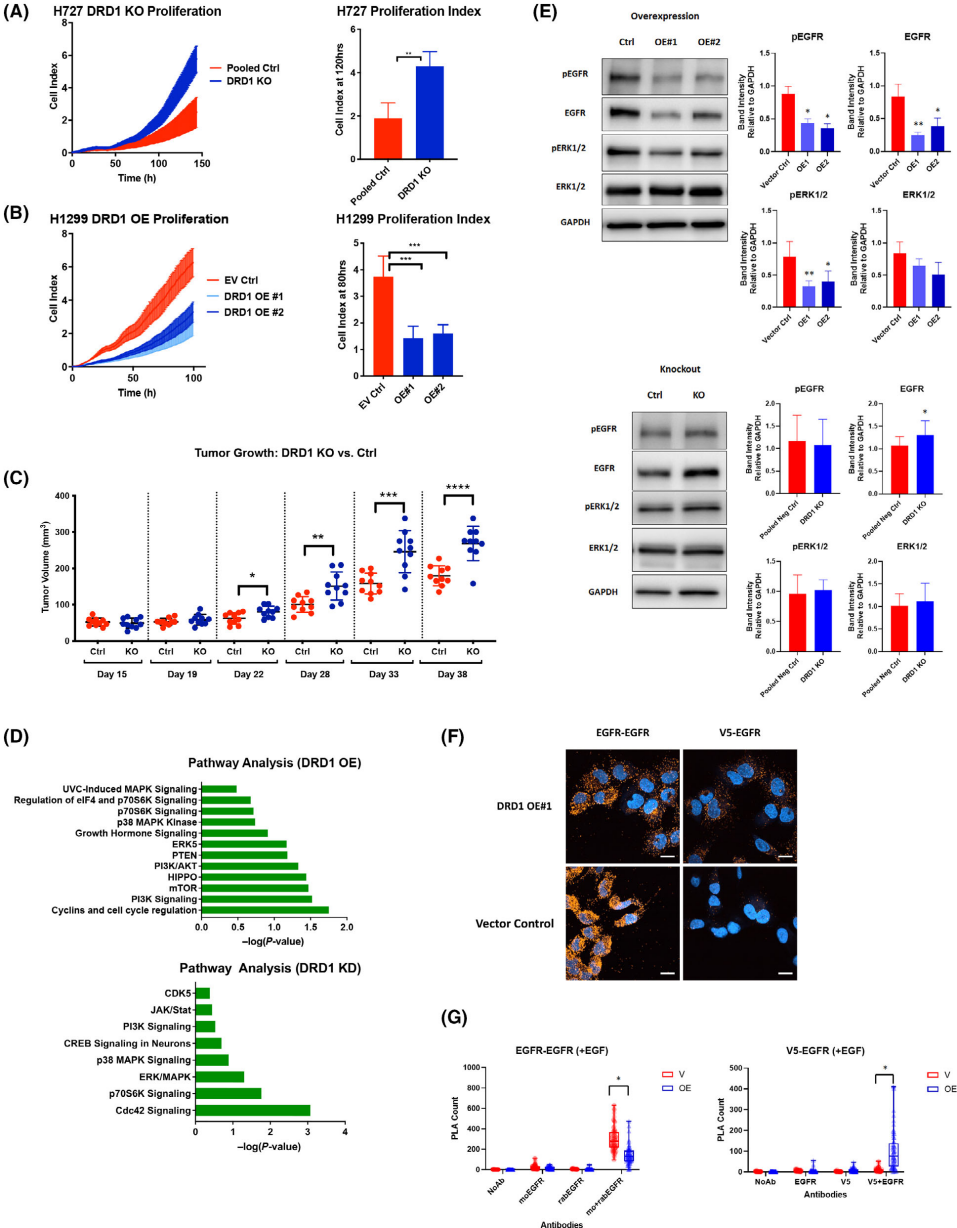
DRD1 inhibits cell proliferation through EGFR. (A) Cell proliferation assay using xCELLigence real‐time cell analysis system comparing growth of H727 DRD1 knockout (KO) cells and control cells (*n* = 4, two‐tailed *t*‐test, **P < 0.001). (B) Cell proliferation assay using xCELLigence real‐time cell analysis system comparing growth of H1299 DRD1 overexpression (OE) cells and empty vector (EV) control cells (*n* = 4, ordinary one‐way ANOVA with Dunnett's multiple comparisons test, ***P < 0.0001). (C) Tumor growth volume over time following subcutaneous injection of 1 × 10^5^ H727 DRD1 KO cells and 1 × 10^5^ H727 Pooled Control (Pooled Ctrl) cells in the left and right flanks, respectively. Graph shows mean ± SD for *n* = 10 carrying one of each *DRD1* genotype (two‐tailed *t*‐tests, *P < 0.05, **P < 0.01, ***P < 0.001, ****P < 0.0001). (D) Ingenuity pathway analysis following transcriptomic analysis of H1299 DRD1 OE cells and H727 DRD1 knockdown (KD) cells compared to respective controls. (E) Representative images of western blot analysis, accompanied by graphs of densitometry analysis, of EGFR pathway proteins in two H1299 DRD1 OE cell lines (*n* = 3, two‐tailed paired *t*‐tests, *P < 0.05, **P < 0.01 compared to vector control) and H727 DRD1 KO cells (*n* = 8, two‐tailed paired *t*‐test, *P < 0.05 compared to pooled control). (F) Representative images of proximity ligation assay showing proximal interactions (PLA signals) between EGFR and EGFR (assay positive control) and between V5‐tagged DRD1 and EGFR in H1299 cells. Images representative of three independent experiments. Scale bars = 20 μm. (G) Quantification of V5‐EGFR and EGFR‐EGFR PLA signals in H1299 vector (V) and DRD1‐expressing (OE) cells, shown as box‐and‐whisker plots of results pooled from three independent experiments with at least 20 cells quantified per experiment per group (*P < 0.0001, Mann–Whitney U, two‐tailed). Bar graphs in panels A, B, and E show mean + SD.

To understand the molecular mechanisms linking DRD1 with lung cancer, we generated stable cell lines harboring CRISPR‐mediated knockout of DRD1, shRNA‐mediated stable DRD1 knockdown, or stable induced overexpression of V5‐tagged DRD1. DRD1 knockout and knockdown experiments were performed in H727 and A549 cells, two cell lines with relatively high levels of endogenous DRD1 expression, and induced DRD1 expression was performed in H1299 and H292 cells, two cell lines with very low endogenous DRD1 expression (Fig. 2A,B, Fig. S3A–F). Finally, to address the possibility that a clonal effect was causing differences in cell proliferation, we also used siRNA to transiently knockdown DRD1 in H727 cells (Fig. S4D,E). In culture, we observed significant differences in cell growth, quantified using xCELLigence. CRISPR‐mediated knockout of DRD1 in lung cancer cell lines significantly increases cell proliferation (Fig. 2A, Fig. S3A–C), as does shRNA‐mediated stable DRD1 knockdown (Fig. S4B,C). Transient siRNA knockdown of DRD1 also increases cell proliferation (Fig. S4D,E), validating that this effect on proliferation is unlikely to be the result of single cell clone selection. Conversely, overexpression of DRD1 in cell lines lacking DRD1 expression suppresses cell growth (Fig. 2B, Fig. S3D–F). Consistent with this trend, we also found that treating H727 cells with the selective DRD1 agonist, SKF‐38393, leads to significant reduction in cell proliferation while treatment with the selective antagonist, SCH‐23390, leads to a significant increase in cell proliferation (Fig. S4A).

Further, we verified this suppressive effect on cell growth in a xenograft model (Fig. 2C, Fig. S4G). In addition to observing significantly faster cell growth *in vitro*, *DRD1* KO tumors grew significantly faster than wild‐type *DRD1* control cells *in vivo* (Fig. 2C, Fig. S4G). Additionally, *DRD1* KO tumors had several histological features consistent with faster tumor growth including a significant increase in the number of observed mitotic figures, increased necrosis, decreased supporting stromal tissue, and decreased tissue differentiation compared with tumors expressing DRD1 (Fig. S4F).

### 3.4 DRD1 modulates the EGFR/MAPK/ERK signaling axis

As little is known about the transcriptional and signaling changes associated with modulation of DRD1 expression outside of the central nervous system, especially in lung tissues, we performed gene expression profiling using Clariom™ S assays (Affymetrix, Thermo Fisher Scientific), along with Ingenuity pathway analysis. A pathway analysis of significantly modulated genes showed expected changes in “dopamine canonical pathways” in both DRD1 downregulated and overexpression cells (Fig. S3I). Additionally, the pathway analysis by both methods showed that DRD1 modulates the MAPK and PI3K signaling pathways in both cell lines (Fig. 2D, Fig. S3I).

To better understand the signal transduction molecules responsible for mediating the effect of DRD1 on cell proliferation and confirm the results of the transcriptomic analysis, kinome profiling was performed on these cell lines using the Human MAPK Cell Profiler. Consistent with the transcriptome data, the phosphoproteins of the MAPK and PI3K signaling pathways were modulated by DRD1 in both the overexpression and downregulated cell lines (Fig. S3G,H). Specifically, we found that MAPK and PI3K pathways are more highly activated in DRD1 shRNA knockdown H727 cells compared with non‐targeted control cells while DRD1 overexpression in H1299 cells decreases MAPK and PI3K pathways activation compared with control cells. Collectively, the transcriptome and kinome analyses suggest that DRD1 reduces the activation of MAPK and PI3K pathways, suggesting a possible mechanism by which DRD1 inhibits cell proliferation.

Interestingly, kinome profiling also indicated that DRD1 reduces EGFR phosphorylation (Fig. S3G,H). To validate these observations, the activation of EGFR and its downstream effectors was characterized in the H1299 and H727 cells with differentially expressed DRD1. Using western blotting, we confirmed that induction of DRD1 inhibits total EGFR expression and phosphorylation, along with reduction in ERK1/2 phosphorylation (Fig. 2E). In contrast, knockout of DRD1 increases expression of EGFR with changes in phospho‐EGFR and phospho‐ERK1/2 that were not statistically significant (Fig. 2E).

Together, our data suggest that DRD1 can reduce the phosphorylation and total expression of EGFR protein in lung cancer cells. However, this effect was not consistently observed at the mRNA level (data not shown). Thus, we investigated the possibility of a direct interaction between DRD1 and EGFR protein. Using proximity ligation assay (PLA), we detected significant proximate interaction (within 40 nm) between V5‐tagged DRD1 and EGFR in the H1299 DRD1 OE cells. Consistent with earlier observations of reduced EGFR expression in the presence of DRD1, we also observed ~45% reduction of EGFR homodimerization in the DRD1 OE cells compared to control cells, consistent with a reduction of EGFR activation and/or expression (Fig. 2F,G).

### 3.5 DRD1 regulates PD‐L1 expression

Given the well‐established link between EGFR and the immune checkpoint molecule, PD‐L1 [42, 43], we next asked if DRD1 could play a role in mediating response to immune checkpoint inhibition (ICI). Currently, tumor expression of PD‐L1 is the primary clinical predictor of patient response to ICI in NSCLC patients. Thus, we first investigated whether DRD1 induction could affect cellular expression of PD‐L1.

**Fig. 3:**
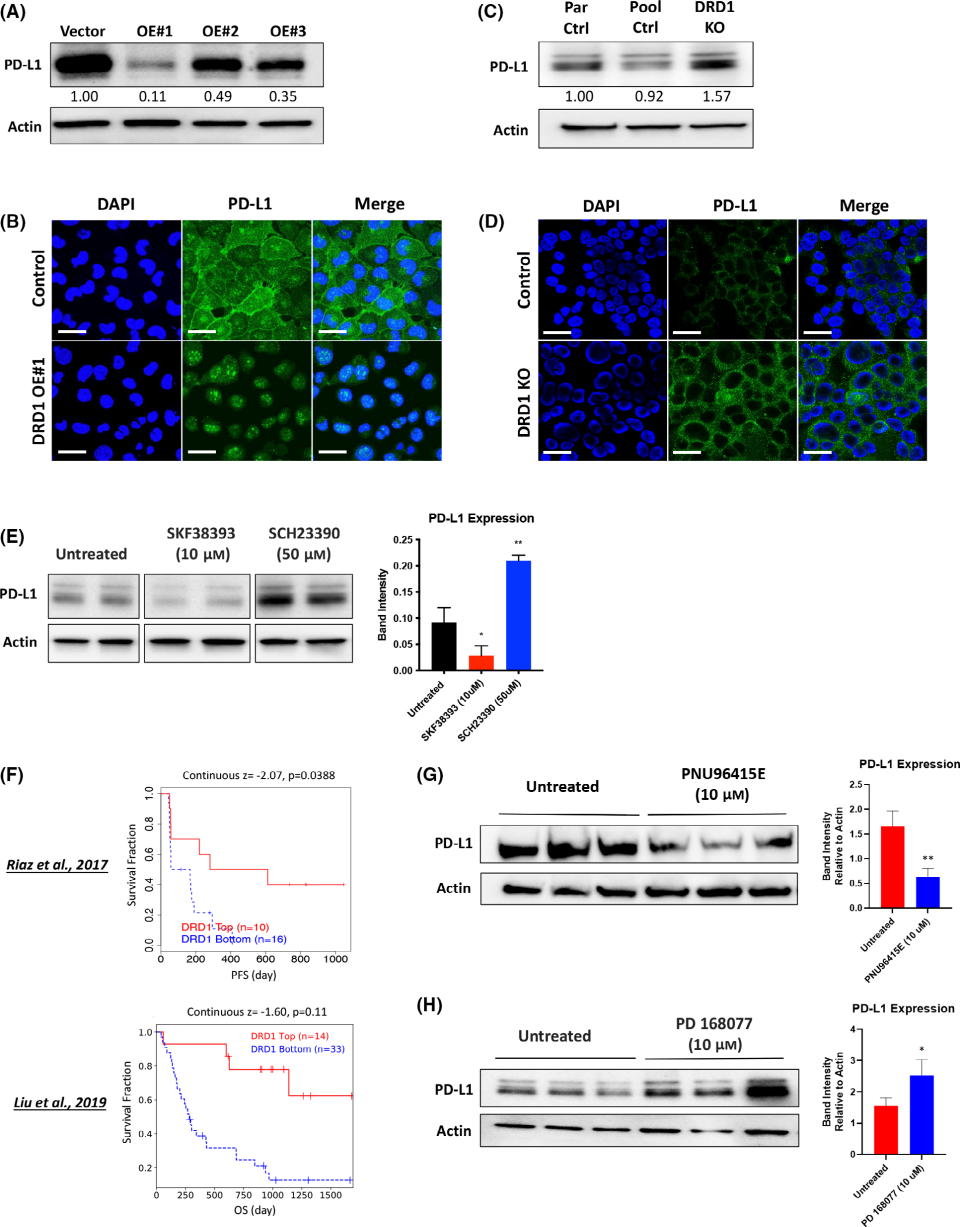
DRD1 modulates PD‐L1 expression in NSCLC cells. (A) Western blot analysis of PD‐L1 expression in DRD1 OE H1299 cells, with 15 μg of total protein loaded for each sample. Blot is representative of three independent experiments. (B) Immunofluorescent staining of PD‐L1 on permeabilized H1299 DRD1 OE cell clones. Images are representative of three independent experiments. Images were captured using confocal microscopy. Scale bars = 50 μm. (C) Western blot analysis of PD‐L1 expression in H727 DRD1 KO cells, with 15 μg of total protein loaded for each sample. Blot is representative of two independent experiments. (D) Immunofluorescent staining of PD‐L1 on non‐permeabilized H727 DRD1 KO cell clones. Images are representative of two independent experiments. Scale bars = 50 μm. (E) Western blot analysis of PD‐L1 expression in H727 cells treated with 10 μm SKF‐38393 or 50 μm SCH‐23390 for 24 h, accompanied by graph results of densitometry analysis of band intensity. (F) Survival analyses of ipilimumab‐pretreated patients treated with second line nivolumab in two clinical trials [49, 50], stratified into DRD1 high or DRD1 low expression groups. Graphs were generated using TIDE. (G) Western blot analysis of H727 cells treated with 10 μm DRD4 antagonist PNU96415E for 24 h, accompanied by graphs of densitometry analysis. (H) Western blot analysis of H727 cells treated with 10 μm DRD4 agonist PD168077 for 24 h, accompanied by graphs of densitometry analysis. Bar graphs in panels E, G, and H show mean ± SD for *n* = 3, and statistical significance was determined using two‐tailed paired *t*‐tests, *P < 0.05, **P < 0.01 compared to respective controls.

We found that cellular PD‐L1 protein expression was significantly reduced between 50% and 90% in H1299 cells expressing DRD1, as visualized by immunofluorescence and quantified by flow cytometry and western blot densitometry (Fig. 3A,B, Fig. S5B,C). Interestingly, PD‐L1 expression was particularly reduced at the cell membrane while PD‐L1 was also observed in the cytoplasm and nucleus in these lung cancer cells, and the nuclear PD‐L1 was not substantially reduced by DRD1 expression (Fig. 3B). An alternative PD‐L1 antibody validated the visualization of the nuclear PD‐L1 (Fig. S5C). We also characterized the expression of PD‐L1 in secreted extracellular vesicles, which are known to be critical in the process of immune evasion in a variety of cancer types [44, 45], and found that DRD1 also reduced exosomal PD‐L1 expression without altering the secretion of total exosomes, quantified using Nanosight software (Fig. S5D). Conversely, cellular PD‐L1 expression was increased by 20–90% in DRD1 KO H727 cells compared to control cells (Fig. 3C,D).

Interestingly, transcription of PD‐L1 mRNA was not consistently reduced by DRD1 overexpression (Fig. S5A). However, this discordance between expression levels of PD‐L1 protein and mRNA is commonly observed in numerous cancer types [46, 47], and it highlights the complex milieu of posttranslational events regulating PD‐L1.

### 3.6 Pharmacological targeting of DRs affects PD‐L1 expression

As there is currently considerable focus on identifying actionable drug targets to synergize with ICI, the relationship between dopamine receptors and PD‐L1 was next investigated in a more physiologically relevant system using the DRD1‐expressing H727 cell line. When these cells were treated with SKF‐38393 (*t* = 24 h), we observed a 50–75% decrease in PD‐L1 protein expression by western blot, consistent with the effect observed in the DRD1 OE H1299 cells (Fig. 3E). Conversely, when these cells were treated with SCH‐23390 (*t* = 24 h), we observed a 25–90% increase in PD‐L1 protein expression by western blot (Fig. 3E).

Earlier, it was demonstrated that DRD1 expression is downregulated in NSCLC patients, suggesting that it could be a challenging drug target. However, we also observed that several D2R family members are overexpressed in NSCLC and other cancer types (data not shown). In broad terms, the D2R family, consisting of DRD2‐4, functions in an opposite manner to the D1R family. Thus, we hypothesized that DRD4 could serve as an alternative target to modulate PD‐L1 expression in an opposite manner than that which was observed with DRD1.

Indeed, we found that the DRD4 antagonist, PNU96415E, reduced PD‐L1 protein expression by western blot (Fig. 3G), consistent with the effects of DRD1 overexpression and agonism. Conversely, we observed that the DRD4 agonist, PD168077, increased PD‐L1 protein expression by western blot (Fig. 3H), consistent with DRD1 knockout and antagonism.

To more directly interrogate the role of DRD1 in mediating patient response to ICI, we queried the Tumor Immune Dysfunction and Exclusion (TIDE) database to harness gene expression data from clinical trials of checkpoint inhibitors [48]. Although there were no suitable datasets collected from lung cancer trials, data from two recent clinical trials of nivolumab in advanced melanoma [49, 50] were used to compare patient response to treatment when patients were stratified by level of DRD1 mRNA expression. Similar to other deleterious effects of reduced DRD1 expression in NSCLC tumors reported in the present study, we found that patients in the DRD1‐low expression groups had significantly worse survival compared those in the DRD1‐high expression groups in both datasets (Fig. 3F). These data provide support for our hypothesis that dopamine signaling is capable of mediating patient response to immunotherapy.

## 4 Discussion

We present evidence that the dopamine receptor family is expressed throughout the lung at the protein level. DRD1 specifically is downregulated in lung cancer, at least partially through methylation of the *DRD1* promoter. Further, we find that DRD1 expression is associated with patient survival with high expression conferring a favorable outcome. Utilizing preinvasive CIS tissue, we also show that aberrant hypermethylation of the DRD1 promoter is an early event in lung cancer, which may be predictive of disease progression in lung CIS patients.

Epithelial cells secrete dopamine at levels comparable with those found in the brain, and to our knowledge, this is the first time these data have been shown. Dopamine is a catecholamine that functions both as a hormone and a neurotransmitter. It is synthesized from the hydroxylation of tyrosine by TH to L‐DOPA and subsequent decarboxylation by DDC. We found both TH and DDC mRNA to be significantly upregulated in lung cancer (Fig. S2J). Currently, the presence of dopaminergic innervation in the human lung is not well characterized. However, a recent murine study demonstrated that sympathetic dopaminergic neurons, defined by TH protein expression, are present and responsible for dopamine secretion in early postnatal lungs, before converting to noradrenergic neurons in adult life [51]. Taken together, this suggests that dopaminergic innervation is unlikely to account for the increased secretion of dopamine observed in adult human lung cancer cells, but instead that it could be due to aberrant upregulation of TH and DDC in lung cancer. Our observation of DRD1 and DRD4 expression in the cilia of bronchial epithelial cells is novel, and while there is no known function of DRD1 in these cilia, we speculate that they may play a role in airway sensing, movement, and clearance.

Our data supported a relationship between improved survival and high DRD1 levels. Analysis of lung cancer data from the Human Protein Atlas (https://www.proteinatlas.org/ENSG00000184845‐DRD1/pathology/lung+cancer) does not replicate that association although when looking at LUAD specifically, there is a trend (*P* = 0.085) for improved survival with high DRD1, and that trend is improved when looking at stages I–II only (*P* = 0.045). Thus, DRD1 levels may be most relevant for early‐stage lung adenocarcinoma, and notably, our analysis of the NCI‐MD study patients focused on stage I LUAD. Interestingly, considering that EGFR mutation is much more common in lung adenocarcinoma than squamous cell carcinoma, our findings linking DRD1 and EGFR signaling suggest that DRD1's regulation of EGFR is a possible mechanism for DRD1 levels being more prognostic in lung adenocarcinoma than squamous cell carcinoma. DRD1 may also be more relevant in certain populations. For example, our NCI‐MD study by design includes a relatively high proportion of African American patients, and such differences in patient population studied could contribute to discrepancies in strength of prognostic marker associations. It is worth further evaluating in what patient subgroups DRD1 can serve as a prognostic marker.

One of the key phenotypes we consistently observed following loss of DRD1 expression in lung cancer cells is increased cell proliferation. This effect was demonstrated *in vitro* and *in vivo* using cell lines where *DRD1* expression was either reduced via shRNA or siRNA or knocked out via CRISPR. The reverse – reduced cell proliferation – was observed with induced DRD1 expression, and similar phenotypic effects were also observed when cell lines were treated with highly selective DRD1 agonists and antagonists. Notably, because there are few NSCLC cell lines with detectable endogenous DRD1 levels (consistent with DRD1 being downregulated in tumor development), we used H727, a bronchial carcinoid cell line, for *in vitro* experiments and recognize this is a limitation of the translatability of our findings to NSCLC. The effects of dopamine receptors on cell proliferation have been reviewed extensively [21] and generally appear to be tissue‐type dependent but with a growing number of tumor types associated with DRD1 as a tumor suppressor [23, 24, 25, 26, 27, 28, 31]. The present study is the first to describe the antiproliferative effects of DRD1 in lung cancer, as well as its mechanism.

The antiproliferative effects of DRD1 appear to be mediated, at least in part, by its modulation of EGFR expression and signaling. Evidence of altered EGFR signaling was supported by phenotypic assays, transcriptome profiling, kinome array analyses, and western blotting in multiple cell lines assessing both the induction and inhibition of DRD1 expression. Our western blotting results for the EGFR‐MAPK pathway confirmed downregulation of EGFR levels and signaling in H1299 cells that had high levels of DRD1 from induced overexpression. Although with DRD1 knockout in H727 cells we only observed an increase in total EGFR without statistically significant increases in EGFR or ERK1/2 phosphorylation, this is unsurprising given the endogenous levels of DRD1 in H727 that are still relatively low, and thus the effect of highly overexpressed DRD1 levels is stronger than the reverse effect of DRD1 knockout.

The mechanism through which DRD1 regulates EGFR remains an open question. The reduction of EGFR expression by DRD1 occurs within the first 4 h of DRD1 agonism, suggesting that the mechanism may be related to posttranslational modification of EGFR or through close interaction between the two receptors, which is supported by our PLA data. As G‐protein‐coupled receptors, D1Rs and D2Rs have been shown to transactivate receptor tyrosine kinases (RTKs) [4]. In particular, DRD1 and DRD2 have been shown to transactivate EGFR in transfected CHO‐K1 cells [52] and primary neuron cultures [53, 54]. DRs can also transactivate other RTKs; for example, DRD1 agonist and antagonist treatment can also affect activation of the RTK TrkB [55]. Alternatively, DRs may also affect RTKs indirectly through non‐canonical G‐protein‐independent signaling via Akt and GSK3 with a variety of effects including RTK activation [4]. Thus, both transactivation and signaling through G‐protein‐independent pathways are possible mechanisms for DRD1's regulation of EGFR signaling, and this warrants further investigation.

EGFR mutations are observed in ~30% of NSCLC patients [56], and considerable efforts have been made to successfully target EGFR and overcome resistance to EGFR‐targeted therapies. Despite their effectiveness, resistance continues to be an issue for all EGFR inhibitors including the most recently approved inhibitor, Osimertinib, with patients achieving a 3‐year survival rate of 28% [57]. Notably this study included only wild‐type EGFR cell lines and did not specifically evaluate EGFR mutant patients, so further studies are needed to elucidate the interactions between DRD1 and EGFR and investigate the potential utility of targeting DRD1 in EGFR mutant NSCLC patients.

As DRD1 has been shown to modulate systemic inflammation via inhibition of the NLRP3 inflammasome in macrophages, we initially tested whether DRD1 modulated inflammasome maturation and cytokine secretion in lung cancer cells, but we did not find evidence for this (data not shown). We did, however, observe that DRD1 regulates PD‐L1 expression in multiple lung cancer cell lines. DRD1 appears to negatively regulate PD‐L1 expression as its overexpression and pharmacological agonism reduced cellular levels of PD‐L1. Conversely, knockout of DRD1 and pharmacological antagonism increased PD‐L1 expression. The precise mechanism by which this regulation occurs should be investigated further in future studies.

The presence of nuclear PD‐L1 in these lung cancer cells was an interesting observation since nuclear PD‐L1 expression has been reported in the literature and has been linked to transcriptomic regulation of immune response genes, as well as other cell autonomous functions related to proliferation and survival [58, 59, 60]. Whether DRD1 induces nuclear translocation of PD‐L1 in cancer cells and induces any downstream signaling effects is an intriguing future direction for DRD1 signaling research. The downregulation of PD‐L1 levels at the cell membrane may also be of clinical significance for synergy with ICI.

The current study is limited in that the reported alterations of PD‐L1 expression following DRD1 modulation were only observed *in vitro* using cultured lung cancer cell lines. More complex *in vitro* co‐culture systems and *in vivo* syngeneic models are required to make further conclusions about the effects of DRD1 on the tumor microenvironment and on the functions of immune effector cells. To determine whether DRD1 modulation does affect the tumor immune microenvironment via PD‐L1, a key next step is to use both wild type and DRD1 knockout immunocompetent animals to evaluate the effects of DRD1‐induced PD‐L1 modulation on tumor‐associated immune cells. DA signaling has previously been shown to regulate CD8 T cell function, as well as the anticancer activity of various other immune cells found in the tumor microenvironment, including macrophages and myeloid‐derived suppressor cells [61, 62, 63, 64, 65]. Thus, there is potential for dopamine pathway‐targeted therapies to synergize with immunotherapies by enhancing antitumor immunity. However, it is currently unknown whether modulating tumor expression of PD‐L1 through pharmacological modulation of DRs would overcome IFN‐γ mediated PD‐L1 induction during the critical process of antigen presentation.

An earlier study in breast cancer showed that chemotherapeutic treatment with etoposide, paclitaxel, and 5‐FU potentiated IFN‐γ mediated induction of PD‐L1 expression, leading to increased T cell apoptosis *in vitro* [66]. In lung cancer, multiple standard‐of‐care platinum‐based chemotherapies have been shown to increase PD‐L1 expression on both tumor cells and immune cells in patient biopsies, and both effects were associated with poorer overall response to therapy [67]. Interestingly, a recent preclinical study investigating the efficacy of the DRD2 antagonist Trifluoperazine (TFP) in colorectal cancer reported that tumors from TFP‐treated mice had increased tumor expression of PD‐L1, as well as increased PD‐1 expression on tumor‐infiltrating CD4+ and CD8+ T cells [68]. Studies such as these highlight the dual action of antineoplastic drugs with their engagement of the immune system in mediating antitumor effects. Thus, the mechanistic underpinnings of dopamine signaling's interaction with immune cell activity and immune checkpoint expression must be interrogated further in order to determine the clinical usefulness of targeting DRs in cancer therapy.

The effects of DR modulation on PD‐L1 observed in this study have led us to hypothesize that DR modulation may provide therapeutic benefit when combined with immunotherapy. One mechanism through which DRD1 agonism and DRD2 antagonism may exert immune‐mediated antitumor effects is that reducing PD‐L1 expression may help overcome immunotherapy resistance in an environment where antigen presentation has already occurred and PD‐L1 mediated T cell exhaustion has promoted resistance. This principle was supported by our observation that high levels of tumor DRD1 expression was associated with increased response to anti‐PD‐1 therapy in two separate cohorts of melanoma patients. Interestingly, in both datasets, this effect was only observed in the ipilimumab‐resistant patients but not in immunotherapy naïve patients, suggesting that higher levels of DRD1 activity provide specific benefit to immunotherapy‐resistant patients (Fig. 3F, Fig. S5E). Although additional studies are necessary before further conclusions can be made, investigating the effect of DR modulation on immune cell functions and its potential combination with immunotherapy modalities are intriguing future directions for this work.

## 5 Conclusions

Here, we show that DRD1 signaling has several anticancer functions that may be targetable in lung cancer patients. DA signaling is present in normal human lung tissue and in lung cancer. Both loss of DRD1 expression and methylation of the *DRD1* promoter are predictive markers of disease progression, and thus, DRD1 promoter methylation may be a negative predictive biomarker in NSCLC patients. DRD1 modulation reduces tumor cell proliferation. Furthermore, DRD1 modulates the expression of EGFR and PD‐L1, two molecules that are key drug targets of FDA‐approved and development‐stage therapies in lung cancer and other cancer types, so targeting DRD1 signaling may also be able to augment these therapies.

## Conflict of interest

The authors declare no conflict of interest.

## Author contributions

CEG contributed to conceptualization, methodology, investigation, validation, writing—original draft preparation, and visualization. ALF and LT contributed to conceptualization, methodology, investigation, validation, visualization, and writing—review & editing. AZ contributed to conceptualization, methodology, validation, and writing—review & editing. ER contributed to software, validation, and visualization. KA contributed to investigation. HT contributed to validation and provision. BMR contributed to supervision and writing—review & editing.

### Peer review

The peer review history for this article is available at https://www.webofscience.com/api/gateway/wos/peer‐review/10.1002/1878‐0261.13608.

## Supporting information


**Fig. S1.** DRD1 and other dopamine pathway proteins are expressed in normal lung tissue.
**Fig. S2.** DRD1 is expressed and its promoter is methylated in malignant lung tissue.
**Fig. S3.** DRD1 inhibits cell proliferation and EGFR signaling.
**Fig. S4.** DRD1 modulates cell proliferation *in vitro* and *in vivo*.
**Fig. S5.** DRD1 modulates PD‐L1 expression.
**Table S1.** Characteristics of cases and controls in NCI‐MD study.
**Table S2.** Characterization of probes used in DRD1 methylation analysis.

## Data Availability

The data that support the findings of this study are available from Dr. ALF (flisal@nih.gov) upon reasonable request.
