# Supplementary material for: DRD1 suppresses cell proliferation and reduces EGFR activation and PD‐L1 expression in NSCLC
Source: Mol Oncol. 2024 Apr 4;18(6):1631–48. doi: 10.1002/1878-0261.13608 (PMC11161724; doi:10.1002/1878-0261.13608)
Supplement: Supplementary file 1 — Fig. S1. DRD1 and other dopamine pathway proteins are expressed in normal lung tissue. Fig. S2. DRD1 is expressed and its promoter is methylated in malignant lung tissue. Fig. S3. DRD1 inhibits cell proliferation and EGFR signaling. Fig. S4. DRD1 modulates cell proliferation in vitro and in vivo. Fig. S5. DRD1 modulates PD‐L1 expression. Table S1. Characteristics of cases and controls in NCI‐MD study. Table S2. Characterization of probes used in DRD1 methylation analysis. [file MOL2-18-1631-s001.pdf]

A

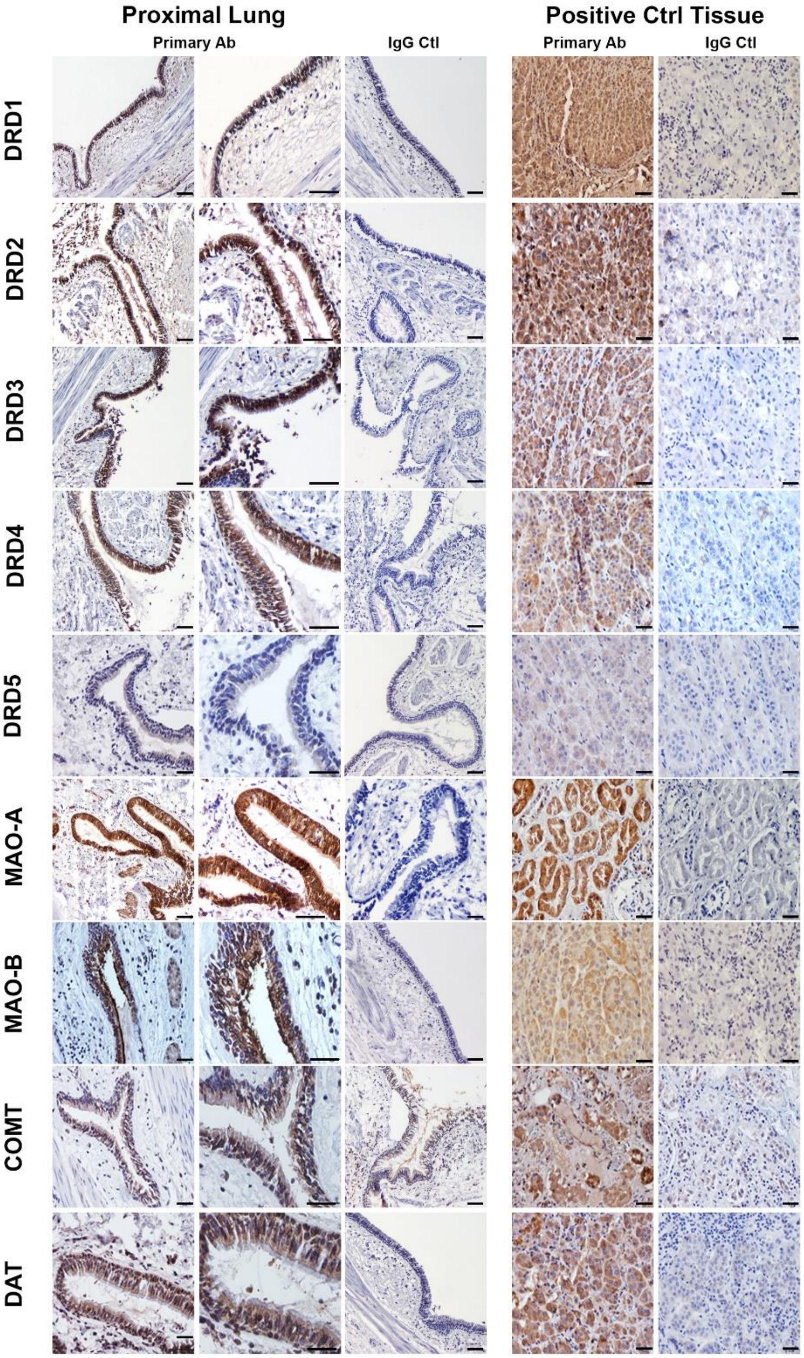
Proximal Lung
Positive Ctrl Tissue
Primary Ab
IgG Ctl
Primary Ab
IgG Ctl
DRD1
DRD2
DRD3
DRD4
DRD5
MAO-A
MAO-B
COMT
DAT

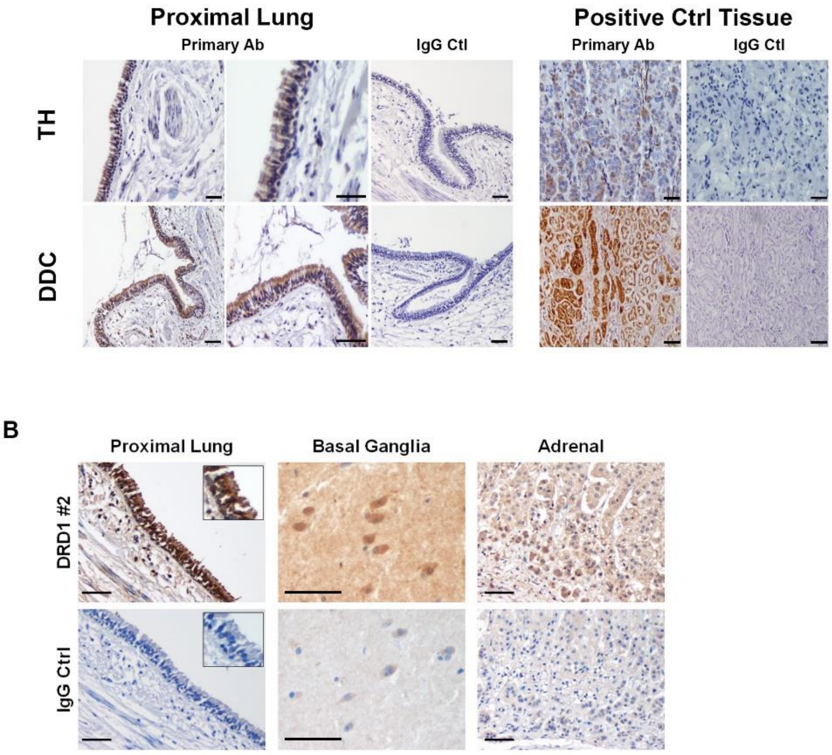

Supplementary Figure 1: DRD1 and other dopamine pathway proteins are expressed in normal lung tissue. A.) Immunohistochemical staining of various DA pathway markers in primary bronchus tissue obtained from non-cancer related autopsy cases. Human adrenal and kidney paraffin sections were used as positive control tissues as recommended by primary antibody manufacturers. Scale bars = 50 µm. B.) Immunohistochemistry for an additional antibody for DRD1 further supports expression of DRD1 in normal human bronchial tissue. Basal ganglia and adrenal gland are used as positive control tissues. Scale bars = 50 µm for images captured at 200x magnification. Enlarged area captured at 400x magnification.

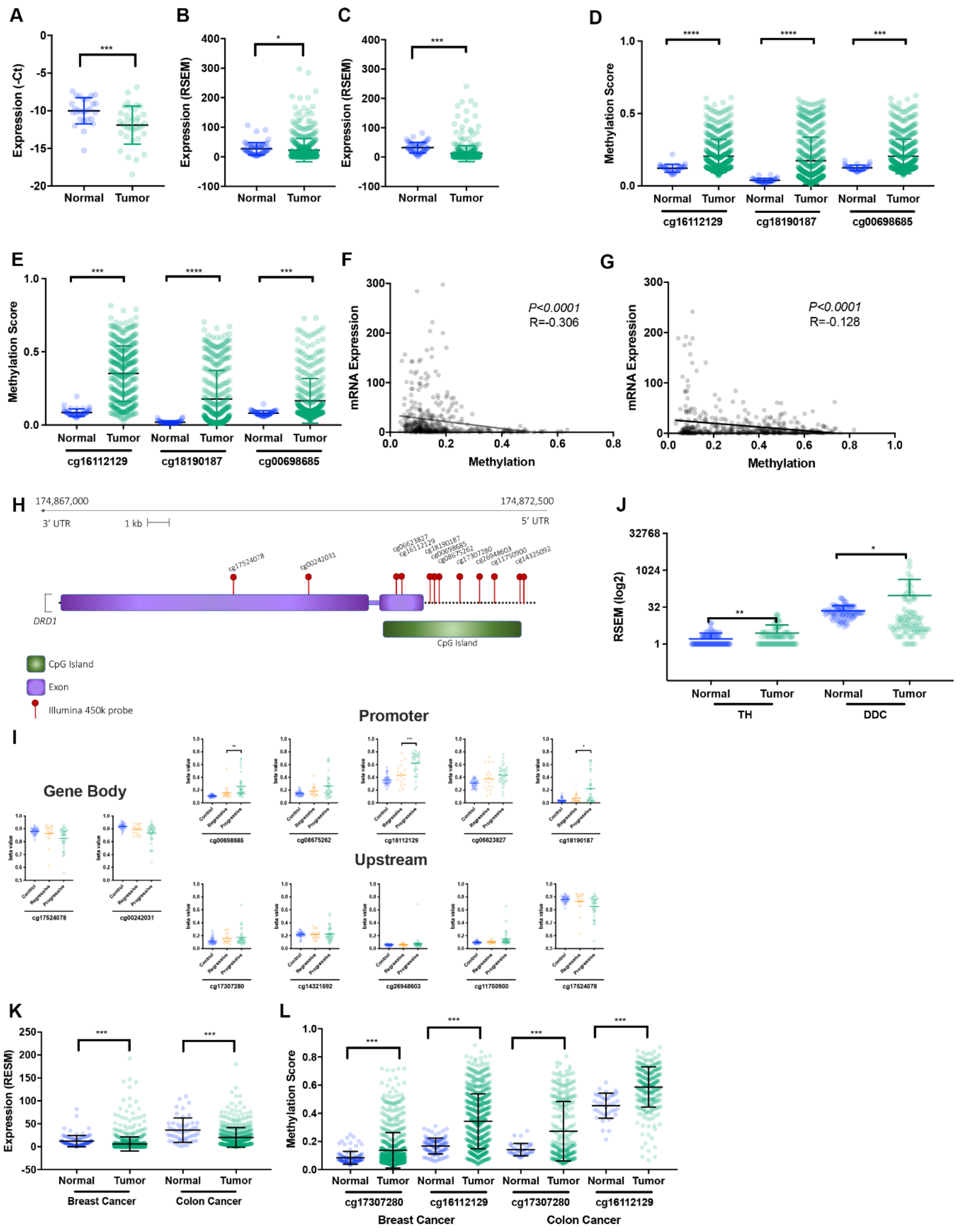

Supplementary Figure 2: DRD1 is expressed and its promoter is methylated in malignant lung tissue. A.) Plot of DRD1 mRNA expression measured by qRT-PCR from NSCLC samples in the NCI-MD cohort. B.) Plot of DRD1 mRNA expression in LUSC patients from the TCGA

dataset. C.) Plot of DRD1 mRNA expression from LUAD patients in the TCGA dataset. D.) Methylation scores (beta values) at three loci proximal to the *DRD1* gene promoter from LUAD patients in the TCGA dataset. E.) Methylation scores (beta values) at three loci proximal to the *DRD1* gene promoter from LUSC patients in the TCGA dataset. F-G.) Scatter plots comparing *DRD1* methylation and mRNA expression levels in LUAD and LUSC, respectively, in the TCGA dataset. H.) Schematic of methylation sites located within the DRD1 gene and upstream region. I.) Methylation scores (beta values) from regressive and progressive lung CIS lesions. J.) Plot of TH and DDC mRNA expression from LUAD patients in the TCGA dataset. K.) DRD1 mRNA expression levels in breast and colon cancer patient datasets obtained from TCGA. L.) Methylation scores (beta values) of DRD1 upstream sites in breast and colon cancer patient datasets obtained from TCGA. Graphs in panels A-E and I-L show mean ± SD. Statistical significance in panels A-E and I-L determined using two-tailed *t*-tests. Significance in panels F-G determined using linear regression. LUAD denotes lung adenocarcinoma, LUSC denotes lung squamous cell carcinoma, and RSEM denotes RNA-Seq by Expectation-Maximization. *p<0.05, **p<0.01, ***p<0.001, ****p<0.0001.

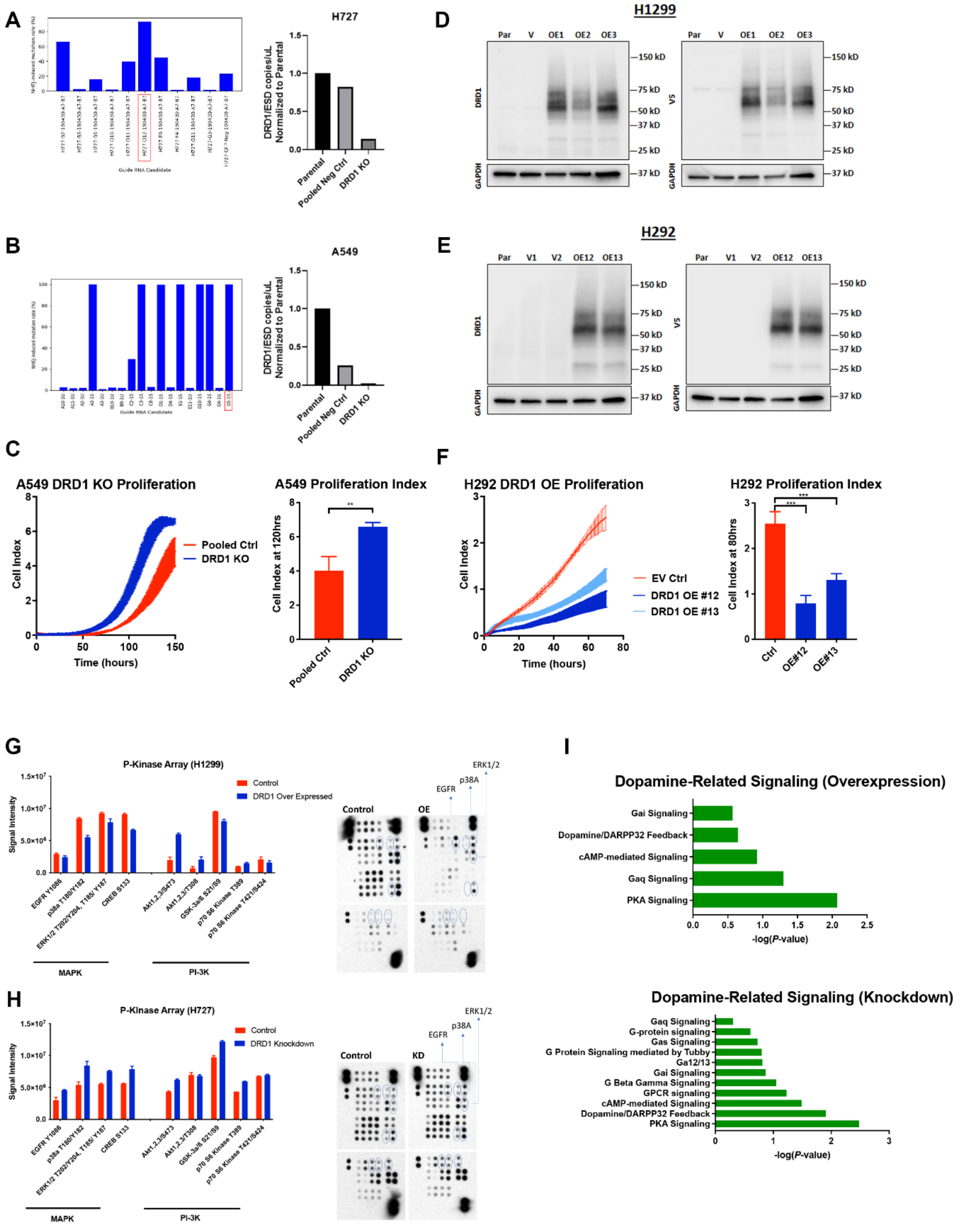

Supplementary Figure 3: DRD1 inhibits cell proliferation and EGFR signaling. A.) Sequencing analysis validating CRISPR editing of the *DRD1* gene in H727 cell clones, accompanied by ddPCR analysis of DRD1 mRNA levels normalized to reference gene ESD in

parental, pooled negative control, and DRD1 knockout (KO) H727 cells. B.) Sequencing analysis validating CRISPR editing of the *DRD1* gene in A549 cell clones, accompanied by ddPCR analysis of DRD1 mRNA levels normalized to reference gene ESD in parental, pooled negative control, and DRD1 KO A549 cells. C.) Cell proliferation assay using xCELLigence real-time cell analysis system comparing growth of DRD1 KO A549 cells with pooled control cells (n=4, two-tailed *t*-test, **p<0.01). D.) Western blot analysis of V5 and DRD1 in H1299 parental (Par), vector control (V), and DRD1 overexpression (OE) clone cell lines verifies induced expression of V5-tagged DRD1. E.) Western blot analysis of V5 and DRD1 in H292 Par, V, and DRD1 OE clone cell lines verifies induced expression of V5-tagged DRD1. F.) Cell proliferation assay using xCELLigence real-time cell analysis system comparing growth of DRD1 OE H292 cells with empty vector (EV) control cells (n=4, ordinary one-way ANOVA with Dunnett’s multiple comparisons test, ***p<0.0001). G-H.) Kinome array results showing differentially phosphorylated proteins in H1299 DRD1 OE and H727 DRD1 knockdown (KD) cells compared to respective controls. I.) Results from IPA of Affymetrix array transcriptomic analysis of H1299 DRD1 OE cells and H727 DRD1 KD cells compared to respective controls.

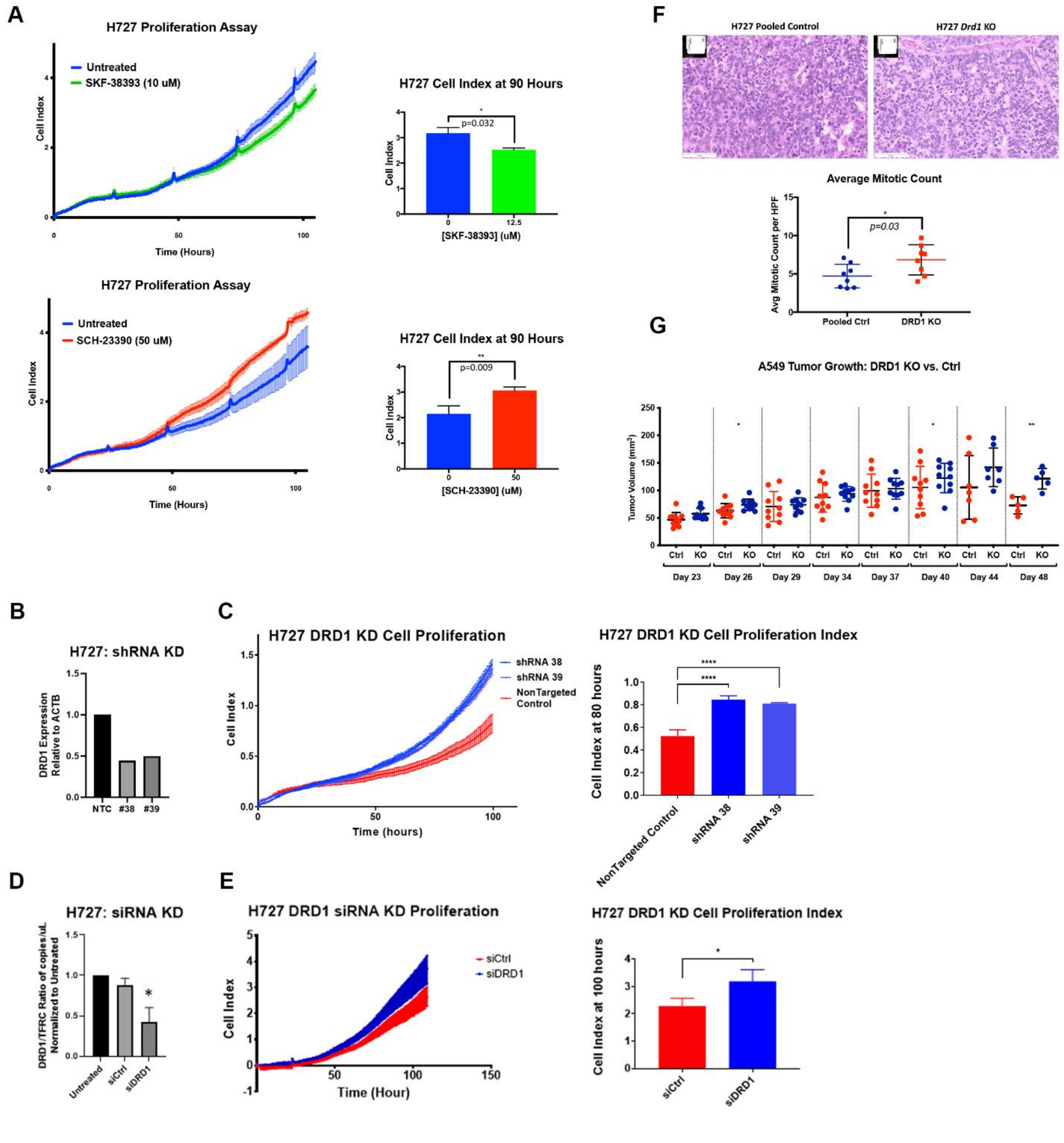

Supplementary Figure 4: DRD1 modulates cell proliferation *in vitro* and *in vivo*. A.) Cell proliferation assay using xCELLigence real-time cell analysis system comparing growth of untreated H727 cells and those treated with SKF-38393 or SCH-23390. B.) RT-PCR analysis of DRD1 mRNA levels normalized to reference gene ACTB showing knockdown of DRD1 in shRNA knockdown (KD) cells compared to control cells. C.) Cell proliferation assay using xCELLigence real-time cell analysis system comparing growth of H727 DRD1 shRNA KD cells and control cells. D.) ddPCR analysis of DRD1 mRNA levels normalized to reference gene

TFRC showing knockdown of DRD1 in siRNA KD cells compared to control cells. E.) Cell proliferation assay using xCELLigence real-time cell analysis system comparing growth of H727 DRD1 siRNA KD cells and control cells. F.) Mitotic figure quantification of H727 DRD1 KO tumors harvested from mice, along with representative images of control and KO tumor histology (n=8). Graph shows mean ± SD. Scale bars = 100 µm. G.) Tumor growth volume over time following subcutaneous injection of $1x10^5$ A549 DRD1 KO cells and $1x10^5$ A549 Pooled Ctrl cells in the left and right flanks, respectively. Graph shows mean ± SD for n=10 carrying one of each *DRD1* genotype. Bar graphs in panels A and C-E show mean + SD. Statistical significance in panels A and C-G determined using two-tailed *t*-tests, *p<0.05, **p<0.01, ***p<0.001, ****p<0.0001.

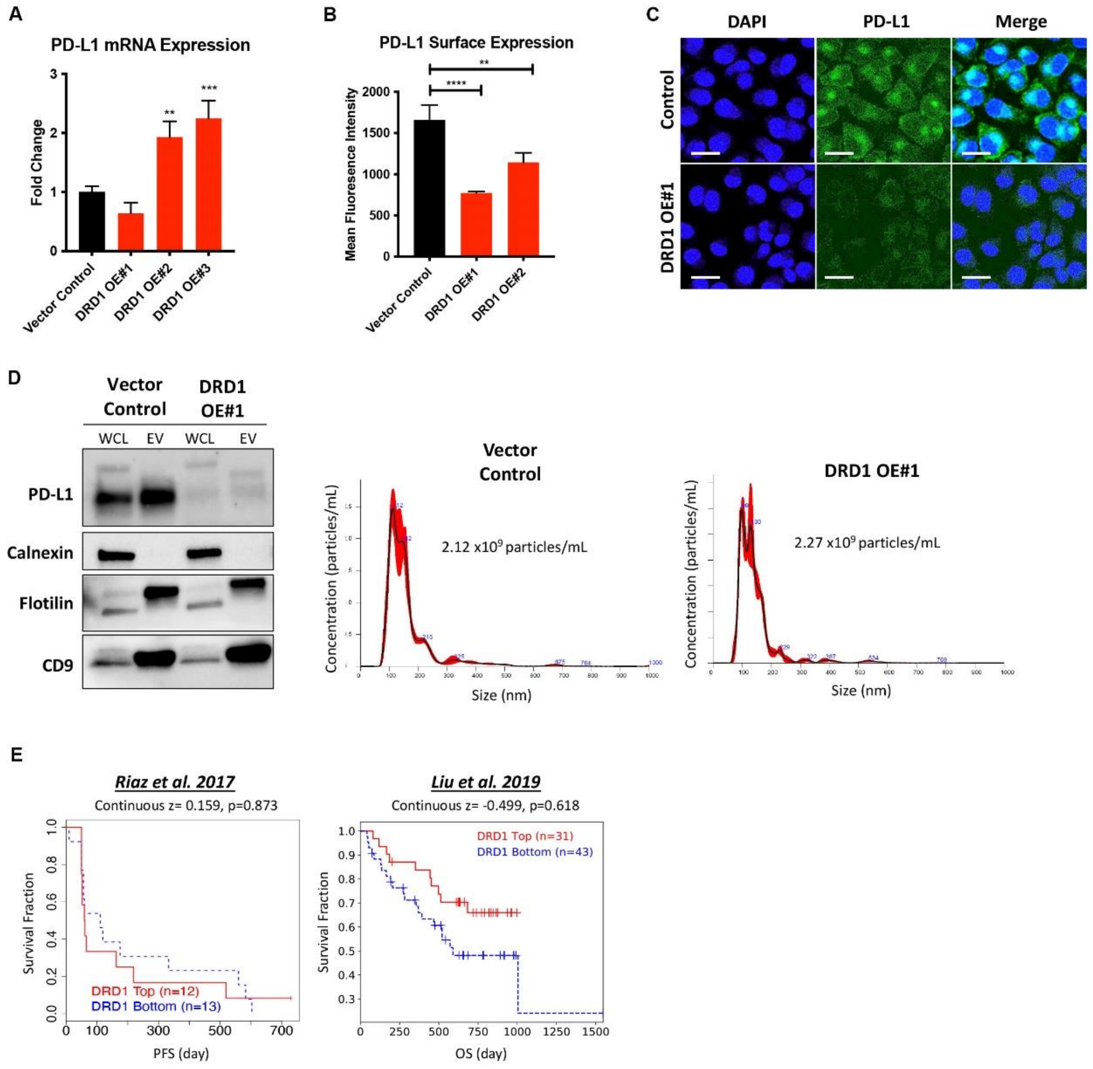

Supplementary Figure 5: DRD1 modulates PD-L1 expression. A.) qRT-PCR analysis of PD-L1 mRNA expression in H1299 DRD1 OE cells. B.) Flow cytometric analysis of PD-L1 expression on the surface of DRD1 OE cells. C.) Immunofluorescent staining of PD-L1 on non-permeabilized H1299 DRD1 OE cell clones using alternative PD-L1 antibody (Abcam, ab205921, clone 28-8). Images were captured using confocal microscopy. Scale bars = 50 µm. D.) Western blot analysis of PD-L1 expression in exosomes secreted by H1299 DRD1 OE cell clones, accompanied by Nanosight characterization of the size and concentration of extracellular vesicles isolated from H1299 cells. E.) Survival analyses of ipilimumab-naïve patients treated with nivolumab in two clinical trials, stratified into DRD1 high or DRD1 low expression groups. Graphs were generated using TIDE. Bar graphs in panels A and B show mean + SD. Statistical significance in panels A and B determined using two-tailed *t*-tests, $*p<0.05$, $**p<0.01$, $***p<0.001$, $****p<0.0001$.

	Controls	%	Cases	%	*P*
Age (mean ± SD)	67.2 ± 8.2		65.7 ± 10.4		0.002
Gender					0.364
Male	398	51.4	326	49.0	
Female	376	48.6	339	51.0	
Smoking status					<0.0001
Never	322	41.7	77	11.8	
Ever	451	58.3	578	88.2	
Race					0.340
African American	314	40.6	253	38.1	
European American	460	59.4	411	61.9	
Pack-years (mean ± SD)	14.5 ± 21.0		39.2 ± 29.5		<0.0001
Tumor histology					
Adenocarcinoma			309	51.8	
Squamous			157	26.3	
Large cell			119	19.9	
Other			12	2.0	

Supplementary Table 1: Characteristics of cases and controls in NCI-MD study (adapted from [1]).

CpG	MAPINFO	Strand	Type	Feature	CGI	UCSC CpG Islands Name	Probe SNPs	Probe SNPs 10
cg00242031	174869880	F	II	Body	shore	chr5:174870753-174872345	rs1799914	rs1042770
cg00698685	174871341	F	II	TSS200	island	chr5:174870753-174872345	rs35916350	
cg01581781	174872371	F	II	TSS1500	shore	chr5:174870753-174872345		
cg06623827	174870895	F	II	5'UTR	island	chr5:174870753-174872345		rs265981
cg08675262	174871399	F	II	TSS1500	island	chr5:174870753-174872345	rs6894164	rs62388321
cg11750900	174872035	F	II	TSS1500	island	chr5:174870753-174872345		
cg14325092	174872330	R	II	TSS1500	island	chr5:174870753-174872345		rs10078866
cg16112129	174870968	R	II	5'UTR	island	chr5:174870753-174872345		rs56000379
cg17307280	174871636	R	II	TSS1500	island	chr5:174870753-174872345		
cg17524078	174869023	R	II	Body	shore	chr5:174870753-174872345		
cg18190187	174871289	R	I	TSS200	island	chr5:174870753-174872345		
cg26948603	174871864	F	I	TSS1500	island	chr5:174870753-174872345		

Supplementary Table 2: Characterization of probes used in DRD1 methylation analysis.

Supporting Information References

1. Robles, A.I., et al., *A DRD1 polymorphism predisposes to lung cancer among those exposed to secondhand smoke during childhood.* Cancer Prev Res (Phila), 2014. **7**(12): p. 1210-8.
